# Curcumin―The Paradigm of a Multi-Target Natural Compound with Applications in Cancer Prevention and Treatment

**DOI:** 10.3390/toxins2010128

**Published:** 2010-01-21

**Authors:** Marie-Hélène Teiten, Serge Eifes, Mario Dicato, Marc Diederich

**Affiliations:** Laboratoire de Biologie Moléculaire et Cellulaire du Cancer, Hôpital Kirchberg, L-2540 Luxembourg, Luxembourg; Email: marie_helene.teiten@lbmcc.lu (M-H.T.); serge.eifes@lbmcc.lu (S.E.); mdicato@gmail.com (M.D.)

**Keywords:** curcumin, cancer, inflammation, cell proliferation, genomic

## Abstract

As cancer is a multifactor disease, it may require treatment with compounds able to target multiple intracellular components. We summarize here how curcumin is able to modulate many components of intracellular signaling pathways implicated in inflammation, cell proliferation and invasion and to induce genetic modulations eventually leading to tumor cell death. Clinical applications of this natural compound were initially limited by its low solubility and bioavailability in both plasma and tissues but combination with adjuvant and delivery vehicles was reported to largely improve bio-availability of curcumin. Moreover, curcumin was reported to act in synergism with several natural compounds or synthetic agents commonly used in chemotherapy. Based on this, curcumin could thus be considered as a good candidate for cancer prevention and treatment when used alone or in combination with other conventional treatments.

## 1. Introduction

Most of the conventional chemotherapeutic agents used today were designed to hit a single intracellular target (e.g., Remicade^®^ to counteract tumor necrosis factor, Avastin^®^ to inhibit vascular endothelial growth factor). Unfortunately, the physiological and mechanistic deregulations responsible for cancer initiation and promotion implicate often hundreds of genes or signaling cascades so that it appears evident that multi-target drugs are requested to overcome complex human diseases such as cancer. Taking advantage of the multiple therapeutic effects observed after the use of natural compounds in traditional medicine, researchers started to evaluate the anti-tumor effect of natural compounds and subsequently tried to understand their mechanism of actions. By this way, they pointed out that curcumin, an active chemical component issued from the plant *Curcuma longa*, exhibits a broad range of activities due to its ability to affect multiple intracellular targets [1]. We will detail hereafter the different intracellular mechanisms affected by curcumin treatment and the resulting therapeutic applications potentially useful for the eradication of cancer.

## 2. Curcumin

**Figure 1:**
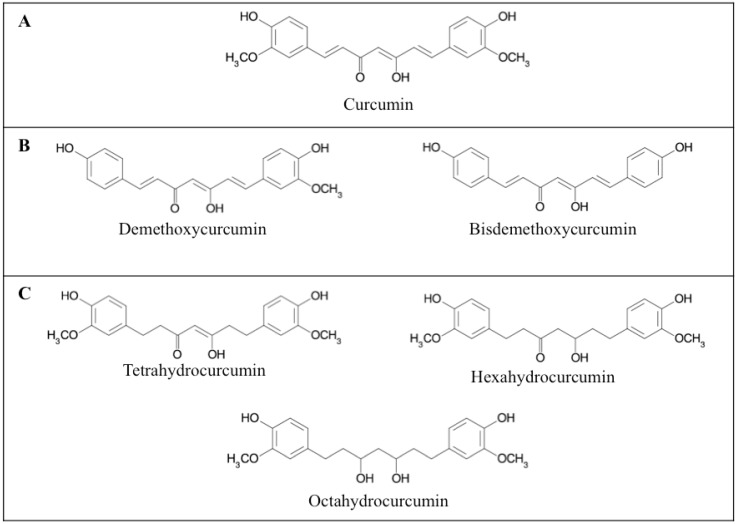
Chemical structure of curcuminoids. Curcumin (A), curcumin demethoxy derivatives (demethoxycurcumin and bisdemethoxycurcumin) (B) and hydrogenated curcumin metabolites (tetrahydrocurcumin, hexahydrocurcumin and octahydrocurcumin).

Curcumin or diferuloylmethane, a polyphenolic molecule extracted from the rhizome of the plant *Curcuma longa*, is a yellow spice that enters into the composition of curry (Figure 1). This natural compound was used over centuries in Ayurvedic, Chinese and Hindu traditional medicine. Nowadays, it appears as a promising chemopreventive compound able to reverse, inhibit or prevent the development of cancer by inhibiting specific molecular signaling pathways involved in carcinogenesis [2,3,4,5]. Whereas clinical trials have already demonstrated the safety of curcumin even at high doses (12 g/day) [6,7,8,9,10], the clinical advancement of this promising natural compound is hampered by its poor water solubility and short biological half-life, resulting in low (micromolar rrange) bioavailability in both plasma and tissues [8,11,12] (Table 1). 

In order to overcome these limitations, several approaches have been tested *in vitro* including the combination of curcumin with adjuvants (e.g., piperine), and the development of delivery vehicles consisting of liposomes, nanoparticules and phospholipid formulations of curcumin. We present in Table 1 the ongoing clinical trials involving curcumin in patients affected by cancer. In these trials, curcumin was mostly administrated alone or in combination with adjuvants (bioperine) or other natural compounds (quercetin, green tea and soybean extracts). Curcumin was also tested for its synergism with conventional treatments such as sulindac, capecitabine and celecoxib (Table 1). Completed clinical trials performed in patients affected by colorectal cancer [9,10,13,14] confirmed the different pharmacological criteria of curcumin cited previously and evaluated the effect of this natural compound on cyclooxygenase (COX-2), leukocytic M_1_G and gluthatione S transferase (GST) levels in patients.

In order to overcome these limitations, several approaches have been tested *in vitro* including the combination of curcumin with adjuvants (e.g., piperine), and the development of delivery vehicles consisting of liposomes, nanoparticules and phospholipid formulations of curcumin. We present in Table 1 the ongoing clinical trials involving curcumin in patients affected by cancer. In these trials, curcumin was mostly administrated alone or in combination with adjuvants (bioperine) or other natural compounds (quercetin, green tea and soybean extracts). Curcumin was also tested for its synergism with conventional treatments such as sulindac, capecitabine and celecoxib (Table 1). Completed clinical trials performed in patients affected by colorectal cancer [9,10,13,14] confirmed the different pharmacological criteria of curcumin cited previously and evaluated the effect of this natural compound on cyclooxygenase (COX-2), leukocytic M_1_G and gluthatione S transferase (GST) levels in patients.

### 2.1. Curcumin analogues and structure related activity

The comparison between the effect of curcumin and its naturally occurring analogues including its demethoxy derivatives (demethoxycurcumin, bisdemethoxycurcumin) and its active hydrogenated metabolites (tetrahydrocurcumin, hexahydrocurcumin and octahydrocurcumin) (Figure 1) pointed out possible structure-activity relationships. 

**Table 1:** Ongoing clinical trials involving curcumin in patients affected by cancer.

**Status**	**Trial name**	**Disease**	**Treatment applied**	**Clinical phase study**
Completed	Curcumin (Diferuloylmethane Derivative) With or Without Bioperine in Patients With Multiple Myeloma	Multiple Myeloma	Curcumin; Bioperine	nd
Recruiting	Curcumin With Pre-Operative Capecitabine and Radiation Therapy Followed by Surgery for Rectal Cancer	Rectal Cancer	Radiation: Radiotherapy; Capecitabine; Curcumin; Placebo	Phase II
Recruiting	Curcumin for Prevention of Oral Mucositis in Children Chemotherapy	Chemotherapy Induced Mucositis	Curcumin liquid extract	Phase III
Recruiting	Curcumin in preventing colorectal cancer in patients undergoing colorectal endoscopy and colorectal surgery	Colorectal cancer	Curcumin	Phase I
			Endoscopy, surgery	
Recruiting	Trial of Curcumin in Advanced Pancreatic Cancer	Adenocarcinoma; Pancreatic Neoplasms	Curcumin	Phase II
Active, not recruiting	Curcumin in Preventing Colon Cancer in Smokers With Aberrant Crypt Foci	Colorectal Cancer; Precancerous/Nonmalignant Condition	Dietary Supplement: curcumin	Phase II
Not yet recruiting	Bio-Availability of a New Liquid Tumeric Extract	Healthy	liquid tumeric/curcumin extract	Phase I
Recruiting	Pilot Study of Curcumin Formulation and Ashwagandha Extract in Advanced Osteosarcoma	Osteosarcoma	Dietary Supplement: Curcumin powder, Ashwagandha extract	Phase I and II
Recruiting	Gemcitabine With Curcumin for Pancreatic Cancer	Pancreatic Cancer	Curcumin (+gemcitabine)	Phase II
Not yet recruiting	Phase III Trial of Gemcitabine, Curcumin and Celebrex in Patients With Metastatic Colon Cancer	Colon Neoplasm	Celecoxib; curcumin	Phase III
Suspended	Curcumin for Treatment of Intestinal Adenomas in Familial Adenomatous Polyposis (FAP)	Familial Adenomatous Polyposis	Dietary Supplement: curcumin; Dietary Supplement: placebo	Phase II
Recruiting	Curcumin for Treatment of Intestinal Adenomas in Familial Adenomatous Polyposis (FAP)	Familial Adenomatous Polyposis	Curcumin	nd
Terminated	Use of Curcumin in the Lower Gastrointestinal Tract in Familial Adenomatous Polyposis Patients	Familial Adenomatous Polyposis	curcumin	Phase II
Recruiting	Phase III Trial of Gemcitabine, Curcumin and Celebrex in Patients With Advance or Inoperable Pancreatic Cancer	Pancreatic Cancer	Gemcitabine; Curcumin; Celebrex	Phase III
Completed	Curcumin for the Prevention of Colon Cancer	Colorectal Cancer	Dietary Supplement: curcumin	Phase I
Terminated	The Effects of Curcuminoids on Aberrant Crypt Foci in the Human Colon	Aberrant Crypt Foci	sulindac; curcumin	nd
Not yet recruiting	A Nutritional Supplement Capsule Containing Curcumin, Green Tea Extract, Polygonum Cuspidatum Extract, and Soybean Extract in Healthy Participants	Healthy, no Evidence of Disease	Dietary Supplement: curcumin/green tea extract/Polygonum cuspidatum extract/soybean extract capsule	nd
Suspended	Sulindac and Plant Compounds in Preventing Colon Cancer	Colorectal Cancer	Dietary Supplement: curcumin, rutin	nd
			Drug : quercetin, sulindac	
Recruiting	Curcumin for the Chemoprevention of Colorectal Cancer	Adenomatous Polyps	Curcuminoids	Phase II
Not yet recruiting	Trial of Curcumin in Cutaneous T-cell Lymphoma Patients	Cutaneous T-Cell Lymphoma	Dietary Supplement: Curcumin (Turmeric)	Phase II

The table was generated by using the registry of federally and privately supported clinical trials conducted in the United States and around the world (http://clinicaltrials.gov). Nd: non defined.

It was reported that the high anti-inflammatory and anti-tumoral potential of curcuminoids are related to low level of hydrogenation and high level of methoxylation but also to the high level of unsaturation of the diketone moiety [15]. On the other hand, the radical scavenging potential of the curcuminoids was linked to the number of *ortho*-methoxy substitutions and to the level of hydrogenation of the heptadiene moiety of curcumin [16,17]. Indeed, glycosylation of curcumin aromatic ring provides a more water-soluble compound with a greater kinetic stability and a good therapeutic index [18].

### 2.2. Curcumin formulations

Due to the multiple therapeutic potential of curcumin, various formulations were tested in order to enhance its bioavailability and to bring this natural compound to the forefront of therapeutic agents [19]. On one hand, formulation of curcumin in nanoparticles [20,21], liposomes [22,23], micelles [24] and phospholipid complexes decreases its hydrophobicity and increase its circulation time, its permeability through membrane barriers, its solubility as well as its resistance to metabolic stress [11]. On the other hand, the use of an adjuvant like piperine isolated from black pepper (that inhibits UGTs and p450s) [25], quercetin derived from soy beans (that inhibits sulfotransferases) and genistein (that inhibits alcohol dehydrogenase) counteracts detoxification enzymes implicated in curcumin metabolism. This leads to the increase of curcumin absorption, serum concentration and subsequently to the increase of curcumin bioavailability [19,26].

## 3. Signaling Pathways Affected by Curcumin Treatment

As cancer is a multifactorial disease, it requires treatment of multiple molecular targets compounds linked to chemoprevention, treatment and drug resistance often observed after chemotherapy. 

**Figure 2:**
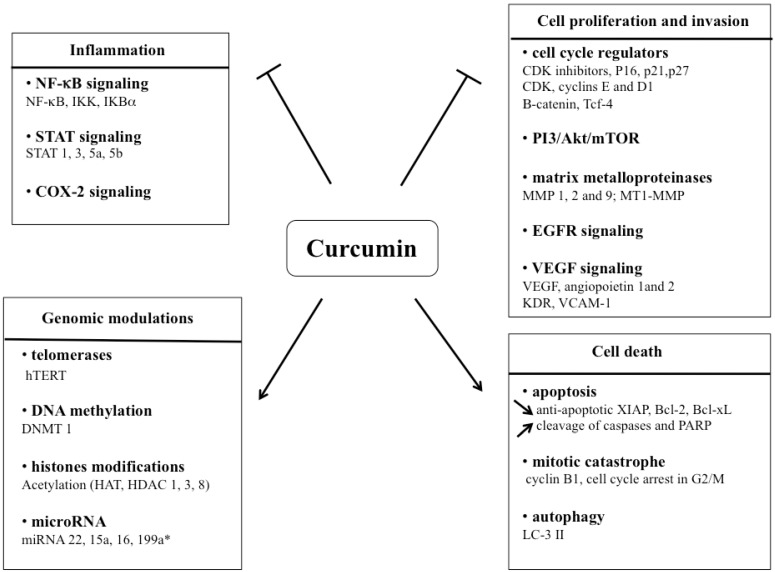
Modulation of multiple molecular targets by curcumin in cancer cells. These modulations lead to initiation and progression steps of carcinogenesis but also to cancer cell death. Arrows represent induction/activation whereas blunt-ended lines represented inhibition/repression.

In an attempt to improve understanding of the pleiotropic targets of curcumin, different studies have been performed so far by microarray gene expression profiling in various cancer types (Table 2). In fact, this technical approach allows to assess simultaneously the expression pattern of a high number of genes at the RNA level [27,28]. Curcumin could effectively be considered a good candidate for cancer prevention when used alone and for cancer treatment in combination with other conventional therapies as it is able to target multiple signaling pathways implicated in this disease [29,30] (Figure 2).

**Table 2:** Evaluation by gene expression profiling of the molecular targets of curcumin in cancer cells.

**Platform**	**Biological System**	**Results**	**Reference**
Oligonucleotide arrays	human K562 chronic myelogenous leukemia cell line	Regulation of cell cycle, JAK-STAT signaling pathway and heat shock related genes by curcumin in TNF-treated K562 cells.	[27]
Oligonucleotide arrays	human BxPC-3 pancreatic carcinoma cell line	Curcumin alters miRNA expression in human pancreatic cells by up-regulating miRNA-22 and down-regulating miRNA-199a*.	[28]
Superarray	human SK-N-MC neuroblastoma cell line	Curcumin is a potent radiosensitizer that inhibits growth of human neuroblastoma cells and downregulates radiation-induced pro-survival factors implicating NF-kB transcription factor.	[29]
Affymetrix	human MDA-MB-231 estrogen-negative breast cancer cell line	Curcumin is able to downregulate the expression levels of inflammatory cytokines CXCL1 and -2 in breast cancer implicating NF-κB transcription factor.	[30]
cDNA arrays	human CL 1-5 lung adenocarcinoma cell line	Curcumin supresses cancer cell proliferation and invasion in lung carcinoma cells by downregulating the expression of MT1-MMP, NCAM, TOPO-I TOPO- II and AXL and the activity of MMP2 and NF-κB. Additionally expression of different HSP family members was induced by curcumin.	[31]
cDNA arrays	human SW620 and Caco-2 colon adenocarcinoma cell lines	Curcumin induces a G2-M cell cycle arrest in epithelial colorectal carcinoma by modulating genes implicated in cell cycle progression	[32]
Affymetrix	wild-type C57BL/6J mice, Nrf2 knockout C57BL/6J/Nrf2(-/-) mice	Novel curcumin-regulated Nrf2-dependent genes implicated in the chemopreventive effects of curcumin in mice liver and intestine were identified. These genes are implicated in ubiquitination, proteolysis, electron transport, detoxification, transport, apoptosis, cell cycle, cell adhesion as well as kinase/phosphatase and transcription factor activity.	[33]
Affymetrix	human MDA-1986 oral squamous carcinoma cell line	Several putative, novel molecular targets of curcumin were identified, amongst which ATF3, a contributor to the proapoptotic effects of this compound.	[34]
Illumina	human HF4.9 follicular lymphoma cell line	Curcumin is able to downregulate CXCR4 and CD20 in follicular lymphoma cells. These genes play an important role in pathogenesis of follicular lymphoma.	[35]
cDNA arrays	human RKO adenocarcinoma cell line	Curcumin downregulates p53 target genes at the RNA level. This effect is mediated by disrupting the native conformation of wild-type p53 protein.	[36]
cDNA arrays	human HT29 colon adenocarcinoma cell line	Confirmation of the known effects of curcumin as cell cycle arrest in G2/M arrest and induction of phase-II genes). Extension of the existing knowledge on these physiological effects and detection of new mechanistic impact such as its effects on tubulin genes and the differential expression of p16(INK4), p53 and RB1.	[37]
Affymetrix	rat C6 glioma cell line	Four primary pathways are targeted by curcumin in neuroglial cells, including oxydative stress, cell cycle control, DNA transcription and metabolism. Additionally new target genes related to oxidative stress as well as cell cycle control were identified.	[38]
Affymetrix, Superarray	human LNCap androgen-responsive prostate adenocarcinoma cell line, human C42B androgen non-responsive prostate adenocarcinoma cell line (derived from LNCap cell line)	Oxidative stress response was identified as the major pathway involved in curcumin induced biological responses in prostate cancer cells. Additionally curcumin suppresses androgen receptor in androgen responsive and refractory cells.	[39]

### 3.1. Curcumin and inflammation

Over the past decades, experimental studies were performed to understand the intracellular mechanisms targeted by curcumin that implicated in the promising therapeutic potential of this natural compound used since centuries in traditional medicine. Aggarwal and coworkers firstly described curcumin as a potent modulator of inflammatory cell signaling [31]. This finding was promising as inflammation is highly involved in the promotion step of tumorigenesis, through the induction of survival, proliferation, invasion, angiogenesis and metastasis [32]. Aggarwal pointed out that this effect on inflammation was mainly due to inhibition of the nuclear factor-kB (NF-κB) signaling pathway [33]. Subsequent studies revealed that this natural compound acts on several components of this pathway. In fact, curcumin was shown to suppress the activation of IkBa kinase (IKK), the phosphorylation and degradation of IkBa and the subsequent phophorylation and nuclear translocation of the p65 subunit in several cancer and premalignant cell types [34,35,36,37,38,39,40]. This prevention of NF-κB activation is also related to the ability of curcumin to inhibit the proteasome function [41,42,43]. Such an inhibition of NF-κB was also induced after treatment with curcumin analogs such as demethoxycurcumin, bisdemethoxycurcumin, tetrahydrocurcumin and 3,5-bis(2-flurobenzylidene)-piperidin-4-one (EF24) [16,44]. Similar results were also observed in primary cells issued from multiple myeloma patients [45] and in patients with advanced pancreatic cancer [46]. It is important to notify that NF-κB regulates the expression of more the 450 genes implicated in all main signaling pathways [47] such as tumor cell proliferation (cyclins), invasion potential (matrix metalloproteinases (MMP), adhesion molecules), angiogenesis (vascular endothelial growth factor (VEGF)) [48] but also growth factors (epidermal growth factor (EGF), tumor necrosis factor (TNFa)) and most of the anti-apoptotic genes (Bcl-2 and X linked inhibitor of apoptosis (XIAP)) [49] as well as numerous oncogenes [50]. The down-regulation of the NF-κB pathway by curcumin could thus impact other related signaling pathways as demonstrated in several studies [51,52,53,54,55] and provide opportunities for both prevention and treatment [56].

In addition to its impact on NF-κB, curcumin also affects other molecular events implicated in inflammation and subsequent tumor promotion [31,57] such as inflammatory cytokines (TNFa, interleukines IL-1, IL-6 and IL-8) [58,59], inflammatory transcription factors (STATs), and inflammatory enzymes (Cyclooxygenase (COX)-2, 5-lipoxygenase (LOX)) [60].

It is reported in the literature that persistent activation of STATs also mediates tumor-promoting inflammation through their collaboration with other transcription factors [31,61,62]. Again, the inhibition of this transcription factor represents a promising tool both for prevention and therapy. We reported that curcumin alone inhibits STATs expression, especially the decrease of nuclear STAT-3, -5a and -5b, without affecting neither STAT1 nor the phosphorylation state of STAT1, -3 or -5 in human chronic K562 leukemia cells. When used as a pre-treatment, curcumin inhibits interferon-gamma-induced phosphorylation of nuclear STAT1 and -3 [63,64]. Similar suppression of STAT3 activation was also observed in Hodgkin’s lymphoma [52], T-cell leukemia [65], head and neck squamous cell carcinoma [66], multiple myeloma cells [52,67] and in CD138^+^ cells derived from multiple myeloma patients [45] following curcumin treatment but also after treatment with curcumin analogs such as GO-Y030 [68], FLLL1 and FLLL12 [69] or a curcumin-phospholipid complex [70]. The interferon-a-induced activation of STAT1 was also inhibited by curcumin in human lung A549 carcinoma and melanoma cells [71]. This inhibition of STAT1 as well as the inhibition of NF-κB were suggested to be implicated in the inhibition of COX-2, an important reactive-oxygen-generating enzymes implicated in inflammatory processes [55,72,73,74]. In fact, it was reported that curcumin is a potent inhibitor of COX-2 in several cancer types [37,73,75,76,77,78,79,80,81]. Moreover, studies performed on mononuclear cells from peripheral blood of patients with pancreatic cancer [46] and on oral premalignant cells [40] reported an inhibition of COX-2 expression after curcumin treatment. More recently, fluorocurcumin, a curcumin analog presenting a higher bioavailability than curcumin, was also shown to down-regulate NF-κB and prostaglandin (PG)E2 level so that it was suggested to be a potential agent against COX-2 overexpressing tumors [82]. All of these *in vitro* as well as preclinical studies suggested that targeting components of the inflammatory pathways provide good opportunities for prevention and therapy of cancer [56]. 

### 3.2. Impact of curcumin on tumor cell proliferation and invasion

Carcinogenesis is a multistage process with three successive steps, initiation, promotion and progression. This process is often linked to oxidative stress, chronic inflammation and hormonal imbalance. The chemopreventive effect of curcumin is mainly based on its effectiveness to inhibit tumorigenesis through the decrease of cancer cell proliferation. 

A way for curcumin to counteract cancer cell proliferation consists in the arrest of the cell cycle. This antiproliferative effect was observed in several cancer cell types (prostate, lung, breast and head and neck cancer but also lymphoma and leukemia). In fact, curcumin induces the expression of cyclin-dependent kinase (CDK) inhibitors p16, p21 and p27, and inhibits the expression of cyclin E and cyclin D1 as well as the hyperphosphorylation of retinoblastoma (Rb) protein. This leads to the disruption of cell cycle and to the death of cells by apoptosis [83,84,85]. 

The modulation of cyclins could be related to the impact of curcumin on the Wingless (Wnt) signaling pathway [86,87], especially through the modulation of the b-catenin/T-cell factor (TCF)/lymphoid enhancer factor (LEF) as observed in osteosarcoma [88], colon cancer cells [89,90], breast stem and cancer cells [91,92]. The observed decrease of the b-catenin/Tcf transcriptional activity was due to the decrease of the nuclear level of expression of b-catenin and Tcf-4 [89]. Similar decrease of b-catenin expression was also shown to be responsible of the inhibition of intestinal tumor growth in an animal model of familial adenomatous polyposis [93]. Gene expression profiling by microarray revealed that curcumin was also able to attenuate the expression of Frizzled-1, a Wnt receptor [94]. Demethoxycurcumin and bisdemethoxycurcumin, two curcumin analogs, were also reported to decrease the b-catenin transcriptional activity with a comparable potency as curcumin in colorectal cancer, through the down-regulation of p300, which is a positive regulator of the Wnt signaling pathway. As the tetrahydrocurcumin metabolite exhibits a much lesser impact on the Wnt pathway, it was suggested that the conjugated bonds in the central seven-carbon chain of curcuminoids are essential for the anti-proliferative activity of curcuminoids [95].

Dysregulations of phosphatidylinositol 3-kinase (PI3K)/Akt signaling pathways were also shown to play an important role in cancer cell growth and survival. Thus the targeting of these pathways may provide new anti-cancer strategies [96,97]. Curcumin was reported to be a good inhibitor of phosphoinositol (PI)3/Akt/mammalian target of rapamycin (mTOR) signaling pathway through the modulation of their expression and phosphorylation in a panel of cancer cell lines derived from leukemia [55,98], cervical cancer [99], colorectal carcinoma [100], renal carcinoma [101], breast cancer [102], Ewing’s sarcoma [103], prostate cancer cells and xenografts [104,105,106,107]. This leads to cell death by apoptosis and to the down-regulation of downstream effector proteins and genes such as p53, NF-κB and eukaryotic initiation factors (eIFs) which play an important role in the initiation of protein synthesis [108,109]. Such an inhibition of PI3/Akt/NF-κB signaling pathway by curcumin was also suggested to be implicated in the down-regulation of the expression of P-glycoprotein (P-gp) implicated in the multidrug resistance (mdr) 1b gene-mediated multidrug resistance [110].

Curcumin analogues such as FLLL11, FLLL12 and 4-hydroxy-3-methybenzoic acid methyl ester (HMBME) were also described as good inhibitors of cell proliferation of breast and prostate cancer though the modulation of PI3/Akt/ mTOR signaling [69,100,111].

The proliferation of solid tumors is also highly linked to their potential to establish metastasis and invade surrounding tissues. This phenomenon is based on the ability of tumor cells to produce growth factors such as EGF, VEGF and MMPs.

Matrix metalloproteinases (MMPs) play a major role in this phenomenon by mediating neovascularization, endothelial cell migration and tube formation [112]. The alteration of MMP-1, membrane type-1 matrix metalloproteinase (MT1-MMP), MMP-2, MMP-9 expression was observed after curcumin treatment *in vitro* and *in vivo* in melanoma [113], prostate [114,115], lung [116] and breast cancer cells [117,118]. 

Over-expression of epidermal growth factor receptor (EGFR) is highly implicated in cancer cell proliferation [119,120]. Curcumin was shown to down-regulate the EGFR expression in pancreatic and lung adenocarcinoma expressing COX-2 [81]. This natural compound also inhibits the EGFR intrinsic kinase activity in human epidermoid carcinoma [121], breast [122], prostate [123,124] and colon cancer cells [125]. This decrease of EGFR expression and activity could be subsequent to the inhibition of the ligand-induced activation of EGFR [126], to the peroxisome proliferator-activated receptor-g (PPAR-g) activation [127], to the suppression of EGFR phosphorylation [128] or to the decrease of the early growth factor-1 (Egr-1) transcriptional activity [129] observed after curcumin treatment. A similar decrease of the EGFR expression level was also observed in estrogen receptor negative breast cancer treated with derivatives of curcumin issued from the replacement of phenyl group of cyclohexanone derived curcumin by heterocyclic rings [122].

Curcumin also appears to be a direct *in vitro* and *in vivo* inhibitor of VEGF and fibroblast growth factor (FGF) [48,77,116,130,131,132,133,134], that are usually elevated in many human cancer and that correlate with enhanced microvessel density and metastatic spread. The molecular mechanisms, implicated in curcumin-inhibition of angiogenesis, consist also in the down regulation of the expression of pro-angiogenic components such as *angiopoietin 1* and *2 genes*, Kinase Domain Region (KDR) [131] and cell surface expression of vascular adhesion molecules (VCAM-1) [135]. Curcumin and its metabolite tetrahydrocurcumin were also reported to decrease the microvascular dilatation, tortuosity and hyper-permeability in hepatocellular carcinoma implanted in nude mouse [136].

### 3.3. Curcumin and genomic modulations

Telomeres are specialized heterochromatic structures localized at the end of human chromosomes. They consist of tandem repeats that play structural and functional roles and allow maintaining genome stability. In healthy cells, telomeres are shortened during each cell division so that the cells stop replicating once the telomeres become too short. In contrast, the majority of solid tumors and leukemia acquire elevated telomerase activity to overcome such limitations ensuring thus their immortality and unlimited proliferative potential through the bypass of senescence [137,138]. Telomerase inhibition emerges thus as an attractive target for cancer therapy [139,140]. Treatment of cancer cells with curcumin revealed that this natural compound inhibits telomerase activity by down-regulating the human telomerase reverse transcriptase (hTERT), the catalytic core of telomerase which leads thus to the suppression of cell viability and to the induction of apoptosis [141,142,143,144,145]. Recent findings suggest that such a down-regulation of hTERT could be explained by a decrease of the association of hTERT and p23, a component of the molecular chaperone complex Hsp90-p23 that normally interacts with the rate-limiting catalytic subunit of telomerase [146].

In order to specifically target telomerase, Kappor *et al*., have taken advantage of the RNA subunit conformation of telomerase. They linked a tetraglycine conjugate of curcumin to an 11-mer DNA sequence complementary to a telomerase RNA sequence. Treatment of oral KB cancer cells with such a construct leads to the a significant reduction of cancer cell growth [147].

In addition to genetic alterations, cancer development and progression are also linked to epigenetic modifications [148], which consist mainly in DNA methylation and posttranslational histone modifications (acetylation, methylation, ubiquitylation, phosphorylation and sumoylation). DNA methylation is mediated by DNA methyltransferases (DNMTs). Aberrant hypermethylation of promoter CpG islands of tumor suppressor genes results in their transcriptional silencing and tumor survival [149]. Curcumin as well as tetrahydrocurcumin were reported to block covalently the catalytic thiolate of the C1226 of DNMT1 leading thus to DNA hypomethylation and suggesting a subsequent death of tumor cells [150].

On the other hand, the level of histone acetylation, resulting from the balance of histone acetyltransferase (HAT) and histone deacetylase (HDAC) activities, plays a crucial role in chromatin remodeling and in the regulation of gene transcription [151]. The impact of curcumin on these implicated epigenetic mechanisms was evaluated and revealed that this natural compound is able to induce histone hypoacetylation by blocking the HAT activity *in vitro* and *in vivo* without affecting HDAC [152]. In fact, curcumin was reported to be a potent and specific inhibitor of p300/CREB Binding Protein (CBP) histone acetyltransferase activity in several cancer types by promoting their proteasomal degradation, whereas its tetrahydrocurcumin metabolite does not affect p300 [153,154,155]. Molecular docking carried out for the human HDAC8 enzyme, pointed out that curcumin could also be considered as a potent HDAC inhibitor [156] and other studies showed that it can also significantly decrease the amount of HDAC1 and HDAC3 [157].

More recently, several studies pointed out that deregulation of microRNAs, a category on small noncoding RNAs that function as genes regulators, were also involved in tumorigenesis and related to epigenetics alterations. In fact, the microRNA network is implicated in the regulation of many basic cellular processes such as proliferation, differentiation and cell death [158,159,160,161,162]. Evidence has been provided that dietary compounds such as curcumin could exert their chemopreventive effect through the modulation of miRNAs expression [163]. Indeed, curcumin was reported to alter miRNA expression by up-regulating miRNA-22 and down-regulating miRNA-199a* in human pancreatic cells [164]. On the other hand, this natural compound was also able to up-regulate the expression of miRNA-15a and miRNA-16 which leads to the reduction of the expression of the anti-apoptotic *Bcl-2* gene [165].

## 4. Mechanisms of Cell Death Induced by Curcumin

Curcumin is most often described to induce tumor cell death through the well-described apoptosis process [166]. However, tumor cell resistance to apoptosis appears frequently and is associated with poor prognosis and resistance to cancer treatment [167]. Fortunately, other types of mechanism leading to cell death (e.g., autophagy, mitotic catastrophe) are also induced by curcumin in order to compensate this lack of induction of cell death mechanisms.

### 4.1. Apoptosis

Apoptosis is a tightly regulated mechanism of cell death that can be initiated by intracellular stress signals and extracellular ligands following curcumin treatment in several cancer types [166,168]. The intrinsic induction of apoptosis is triggered in response to cellular signals including stress and DNA damage. Curcumin was reported to induce the up-regulation of pro-apoptotic proteins from the Bcl-2 family (Bim, Bax, Bak, Puma and Noxa) and the down-regulation of anti-apoptotic proteins (XIAP, Bcl-2, Bcl-xL) [101,105]. This leads to the opening of mitochondrial permeability transition pores, the release of cytochrome c, the activation of the apoptosome (caspase-9/apaf-1/cytochrome c) and the subsequent cleavage of caspase-3, -6 and -7, Poly (ADP-ribose) polymerase (PARP) and finally to the death of tumor cells [169,170,171]. Pre-clinical studies pointed out that curcumin has similar pro-apoptotic effect on primary chronic lymphocytic leukemia B cells but that this effect is inhibited in the presence of stromal cells. In order to overcome this protective effect mediated by stromal cells, curcumin needs to be administered simultaneously with epigallocatechin-3 gallate (EGCG) [172]. 

On the other hand, apoptosis may also be induced by the extrinsic pathway at the cell surface through the activation of cell membrane receptors (Fas, TRAIL) [173,174,175]. This pathway leads to the assembly of the death- inducing signaling complex (DISC) containing Fas, FAD and caspase-8 and -10. The extrinsic pathway converges then to the intrinsic one by the induction of Bid cleavage, the subsequent release of cytochrome c and the activation of the cascade of caspases.

This induction of cell death by apoptosis after curcumin or curcumin analogs treatment [176] supports the idea of their possible implication for cancer therapy [16,177]. 

### 4.2. Mitotic catastrophe

Mitotic catastrophe (MC) is a type of cell death resulting from aberrant mitosis. In fact, this process results from a combination of deficient cell-cycle checkpoints (DNA structure and spindle assembly checkpoints) and cellular damage. Moreover, it has been reported that the intrinsic mitochondrial apoptotic machinery (chromatin condensation, mitochondrial release of proapoptotic proteins, caspase-2 activation and DNA degradation) is highly implicated in the execution of mitotic catastrophe, which occurs at the metaphase level, in a p53 independent manner [178,179]. This type of mitosis leads to the formation of large non-viable cells with several micronuclei and uncondensed chromosomes [179,180]. The disruption of mitotic spindle structure and the appearance of micronucleation after curcumin treatment were firstly described in MCF-7 human breast cancer cells and were related to curcumin-induced cell cycle arrest in G2/M [181]. Mitotic catastrophe was also a way for curcumin to overcome the resistance of tumor cells to apoptosis. In the case of curcumin, the induction of MC was related to the inhibition of survivin, a modulator of cell division and apoptosis in cancer [182,183]. Moreover, curcumin-induced cytotoxicity observed in a panel of oesophageal cancer cell lines after 24h of treatment was associated to MC induction related to an accumulation of the mitotic regulator cyclin B1 and of poly-ubiquitinated proteins [184]. Other publications underline the fact that curcumin metabolites, derivatives and products of degradation were unable to induce G2/M cell cycle arrest and MC compared to the original curcumin compound [185,186].

Mitotic catastrophe induction should be considered for clinical approaches, as a correlation was found between poor prognosis of treatment and inability of cells to induce MC. Moreover an increase of MC induction could compensate impaired induction of apoptosis [167].

### 4.3. Autophagy

Autophagy is a highly regulated process characterized by sequestration of bulk cytoplasm, long-lived proteins and cellular organelles in double-membrane vesicles (autophagosomes), which are subsequently degraded in lysosomes. The final role of autophagy as a tumor suppressor or a protector of cancer cells from anti-cancer therapy is still under investigation [187,188,189]. In the case of curcumin, it was recently reported that this natural compound suppresses the growth of malignant gliomas *in vitro* and *in vivo* through cell cycle arrest in G2/M transition phase and induction of autophagy. This phenomenon has been associated to the inhibition of the Akt/mTOR kinase and to the activation of the extracellular signal-regulated kinases (ERK) 1/2 and to the increase of LC-3 II expression [190,191]. Such an induction of autophagy was also observed concomitantly with mitotic catastrophe in oesophageal cancer after curcumin treatment [184].

## 5. Curcumin Synergistic Effect in Combination with Other Natural or Chemotherapeutic Compounds

As described above, curcumin is a multi-target natural chemopreventive compound by itself. In addition to its high chemopreventive potential, this natural compound can act synergistically with other natural compounds or other kinds of therapy (radiotherapy, chemotherapy and hormonotherapy).

### 5.1. Synergism with natural compounds

Curcumin was reported to have a synergistic effect with genistein, a natural compound derived from soy beans. When combined, they are able to reduce the proliferation of the human breast MCF-7 oestrogen-positive cells induced by environmental pesticides [192]. Moreover, combination of curcumin with 1,25-dihydroxyvitamin D3 (calcitrol) treatment, a well known inducer of monocytic differentiation, was able to stimulate monocytic differentiation of the human promyelocytic HL-60 cells [193] and to abolish resistance of calcitrol-differentiated HL60 cells to DNA damage-induced apoptosis by activating other cell signaling pathways leading to cell death [194]. Diet containing both curcumin and omega-3 fatty acids (fish oil diet) leads to the prevention and treatment of pancreatic tumor xenografts through the down-regulation of the expression and activity of iNOS, COX-2 and 5-LOX and up-regulation of p21 [78]. Coadministration of curcumin with embellin, a natural benzoquinone derived from Embelia ribes berries, prevents lipid peroxidation, histological alterations and oxidative tissue damage occurring usually during chemically-induced hepatocarcinogenesis in Wistar albino rats [195]. An additive inhibitory effect on cell proliferation was also observed in human prostate cancer PC-3 cells *in vitro* and *in vivo* in human PC-3 xenografts mice treated concomitantly with curcumin and b-phenylethyl isothiocyanate (PEITC), a natural compound issued from cruciferous vegetables. This inhibitory effect was related to the suppression of epidermal growth factor (EGFR), Akt and PI3K phosphorylations and the inhibition of the NF-κB signaling pathway [128,196]. A similar cascade of events was also pointed out following the combination of curcumin and resveratrol *in vitro* in HCT116 colon cancer cells and *in vivo* in colon cancer xenografts [197]. Synergistic interactions of curcumin with epigallocatechin-3-gallate (EGCG), a polyphenolic compound found in green tea leads to the reduction of their respective dose index in normal, premalignant and malignant human oral epithelial cells [198]. In the case of chronic lymphocytic leukemia cells (CLL), synergism between EGCG and curcumin allows to overcome the stromal-mediated protection of the CLL cells in the case of sequential therapy. By this way, EGCG sensitizes the cells to curcumin effects and is able to increase strongly cell death in leukemic cells [172]. Curcumin administrated in combination with piperine, an alkaloid from black pepper, inhibits breast stem cell self-renewal without inducing toxicity to differentiated cells. This inhibitory effect is mediated by the inhibition of mammosphere formation and Wnt signaling pathway, but without causing toxicity to differentiated cells [91]. 

A clinical approach revealed that the combination of curcumin with quercetin, a plant derived flavonoid, reduces the number and size of ileal and adenomas in patients with familial adenomatous polyposis without appreciable toxicity [199]. Most of the natural compounds cited above to have synergestic effect with curcumin *in vitro* or *in vivo* are included in ongoing clinical trials in patients affected by cancer.

### 5.2. Synergism with conventional therapy

Due to frequent failure of conventional treatment alone (radiotherapy, chemotherapy and hormonotherapy), novel clinical strategies, based on the combination of different treatments together or in conjunction with chemopreventive agents, are emerging. 

The combination of curcumin with radiation enhances significantly the radiation-induced clonogenic inhibition and induces cell death by apoptosis. This combination of treatment reduces the TNFa-mediated NF-κB activity, alters the Bax/Bcl-2 ratio and activates cytochrome c, caspase-9 and caspase-3 in PC-3 prostate cancer cells [200], but also in colorectal cancer cells [53]. Pretreatment of cervical carcinoma cells results in a significant dose-dependent radiosensitization of these cells. This involves an increase of reactive oxygen species (ROS) and extracellular signal-regulated kinase (ERK) 1/2 activation [201]. Altogether these data suggest that curcumin can be considered as a potent radiosensitizer in prostate and cervical cancer.

Several studies have also been performed in order to evaluate the potential synergistic activity of curcumin in combination with synthetic agents commonly used in chemotherapy. We will first focus on the combination of curcumin with chemotherapeutic agents from natural origin and currently used in clinic such as Taxol^®^ (paclitaxel), derived from *Taxus brevifolia*. Curcumin was shown to potentiate the effect of paclitaxel-mediated chemotherapy in advanced breast cancer *in vitro* and *in vivo* and is able to inhibit lung metastasis. Such a sensitization of taxol-induced cell death by curcumin was also observed in cervical HeLa cancer cells. In both case, the potentiation of chemotherapy by curcumin was related to the down-regulation of NF-κB and serine/threonine Akt pathways, to the suppression of cyclooxygenase 2 (COX-2) and matrix metalloproteinase-9 (MMP-9) but also to the activation of caspases and cytochrome c release [117,202,203]. On the other hand, curcumin appears to be a good adjuvant to enhance the induction of apoptosis and the subsequent chemotherapeutic efficacy of vinorelbine for the treatment of advanced non-small lung H520 carcinoma cells [204] and of celecoxib, a well known COX-2 inhibitor, in pancreatic adenocarcinoma cells [205,206]. This synergism of curcumin and celecoxib is responsible for the down-regulation of the COX-2 signaling pathway. Such a reduction of COX-2 is also observed in human colon cancer HT-29 cell lines when curcumin is associated with the antimetabolite 5-fluorouracil (5-FU) chemotherapeutic agent [207]. The combination of curcumin and 5-FU was also reported for the eradication of human gastric adenocarcinoma cells through the induction of cell cycle arrest in G2/M transition phase [208]. Furthermore, curcumin is able to potentiate the antiproliferative effect of 5-FU associated with oxaliplatin or oxoplatin alone and to stimulate cell death by apoptosis through the attenuation of epidermal growth factors (EGF) and insuline-like growth factor (IGF) signaling pathways in colon cancer HCT-116 and HT-29 cells [209,210]. 

In addition to its ability to enhance the antitumoral effect of conventional chemotherapy, curcumin is also able to circumvent chemoresistance. Resistance to conventional chemotherapy often appears in the case of treatment with platinium (Pt) drugs such as cisplatin and oxaliplatin [211,212]. Several reports revealed that combination of curcumin with Pt drugs treatment inhibits the growth of cancer cells by modulating EGF and IGF receptors expression and by inducing G2/M cell cycle arrest through the modulation of Akt and p38 MAPK [209,210,213,214]. This combination also leads to the induction of cancer cell death by apoptosis through the increase of p53 tumor suppressor gene expression, through the proteasomal degradation of Bcl-2 mediating cisplatin resistance and through the reduction of interleukin-6 production [215,216]. In the case of colon cancer, the emergence of a subset of self-renewing cells, called cancer stem cells (CSCs), is highly implicated in the failure of oxaliplatin treatment. Treatment of oxaliplatin resistant CSCs cells with curcumin results in a marked reduction of CSCs cells accompanied by alterations of the level of DNA methyltransferase 1 [217]. These findings underline the fact that curcumin is able to increase the sensitivity of resistant cancer cells to Platinium drugs and to prevent the emergence of chemoresistant colon cancer cells due to the enrichment of cancer stem cells. Sung *et al.*, showed that curcumin overcomes chemoresistance and potentiates the effect of thalidomide and bortezomib by down-regulating NF-κB and its target genes (e.g., cyclin D1, Bcl-xL, Bcl-2, XIAP, survivin and VEGF) in human multiple myeloma *in vitro* and *in vivo* [218]. More recently, curcumin was also found to potentiate the anti-tumor effect of gemcitabine, another pyrimidine antimetabolite, by suppressing proliferative and angiogenic biomarkers and by modulating the NF-κB signaling pathway *in vitro* in human bladder cancer 253JBV cells and *in vivo* in an orthotopic human bladder cancer [134]. Similar results were observed after combination of curcumin with capecitabine in the case of advanced metastatic colorectal cancer in an orthotopic mouse model. This combination was also highly effective in suppressing acites and distant metastasis in other organs [77].

Venkatesan *et al.* also reported that curcumin is able to attenuate the myocardial toxicity usually observed after adriamycin (doxorubicin) treatment of Wistar rats. In fact, such a pre-treatment with curcumin decreases the level of creatinine kinase and lactate deshydrogenase as well as lipid peroxidation and it increases the level of endogenous antioxidants in order to counteract the side effects of anthracycline chemotherapeutic agents [219]. Such combination of treatment could thus limit free radical-mediated organ injury normally observed after adriamycin treatment. 

Curcumin, alone or in combination with docetaxel, was also shown to be highly potent in mice with multidrug-resistant tumors, by decreasing both proliferation, microvessel density and by increasing tumor cell apoptosis [48]. Recently, the results of a phase I dose escalation trial including fourteen patients with advanced or metastatic breast cancer pointed out that curcumin could be used in combination with docetaxel. This study allows to determine the maximal tolerated dose (6 mg/mL curcumin for seven consecutive days every three weeks in combination with a standard dose of docetaxel) as well as their toxicity, safety and effects on tumor marker [220]. Thanks to the encouraging data obtained by clinical trial, a comparative phase II clinical trial study is now under investigation.

Curcumin also appeared to be a good candidate to sensitize prostate cancer cells for TRAIL-mediated immunotherapy. TNFa related apoptosis-inducing ligand (TRAIL) is an inducer of apoptosis in many cancer cells and is an attractive cytokine for the treatment of advanced cancers including prostate cancer. Although prostate cancer cells (DU145, PC-3 and LNCaP) are mostly resistant to TRAIL, they can be sensitized with curcumin to TRAIL-induced apoptosis. This combination induces the inhibition of constitutively active NF-κB, DNA fragmentation, the cleavage of pro-caspase-3, pro-caspase-8 and pro-caspase-9, as well as the truncation of Bid and cytochrome c release. It also leads to the inhibition of the anti-apoptotic proteins Bcl-2, Bcl-xL and XIAP [38,105,221,222,223]. These results were confirmed *in vivo* by pre-clinical studies performed in PC-3 xenografts [224] and in TRAIL-resistant LNCaP xenografts [225].

## 6. The “Dark Side” of Curcumin

All of the previous reported findings have underlined the pharmacologically safety and biological effectiveness of curcumin and analogs. However it appeared that many of the anti-cancer effects of curcumin observed *in vitro* cannot be achieved *in vivo* or in patients mainly due to its low bioavailability outside the gastrointestinal tract after oral administration [9,226]. Moreover, several studies also pointed out that curcumin can exhibit toxicity and carcinogenic effects [12]. In a 2-year study performed on rodents fed with curcumin, an equivocal evidence of curcumin carcinogenic activity was reported as they observed an increased incidence of hepatocellular and clitoral gland adenoma as well as carcinomas of the small intestine, due to curcumin ingestion [227]. Curcumin also appeared responsible for the promotion of lung tumor multiplicity and its progression to later stages in transgenic mouse model expressing the human Ki-ras^G12C^ allele and affected by lung cancer [228]. These negative effects of curcumin were suggested to be mediated by the presence of a,b-unsaturated ketone in its chemical structure that is responsible for the inactivation of the tumor suppressor p53 [229] and the production of ROS [230]. It also implicates its iron chelator potential [231]. Other groups revealed that curcumin could also induce chromosome aberrations [232] and DNA alterations [233], which are both highly implicated in carcinogenesis. Moreover, under specific conditions, curcumin could also be toxic and can alter the efficiency of conventional chemotherapy and radiotherapy [230]. In fact, it has been reported that curcumin inhibits camptothecin-, mechlorethamine-, and doxorubicin-induced apoptosis in human breast cancer *in vitro* by up to 70% and that it also inhibits the cyclophosphamide-induced tumor regression and apoptosis in *in vivo* models in a time and dose dependent manner. It was suggested that this inhibition of chemotherapy-induced apoptosis by curcumin happened through the inhibition of ROS generation and blockade of JNK function after curcumin treatment [234]. All of these findings lead to the conclusion that the balance between risk and benefit of curcumin should be taken into account before any clinical use for cancer prevention or therapy.

## 7. Conclusions

In this review we have summarized the major intracellular components targeted by curcumin treatment. All of these findings reinforce the idea that curcumin could be considered as a potent natural compound both for prevention and treatment of multifactorial disease such as cancer. However, more translational research and clinical trials with either native or formulated curcumin or in combination with compounds already approved for conventional therapies are needed to better understand the benefit-risk profile of curcumin before putting this natural compound in the forefront of clinical cancer therapy. With this in mind, numerous other natural compounds initially used, as curcumin, in traditional cooking or medicine, are now under investigations in order to discover other multipotent natural molecules that could be used in cancer prevention or therapy.
